# Lifetime use of nonsteroidal anti-inflammatory drugs and breast cancer risk: results from a prospective study of women with a sister with breast cancer

**DOI:** 10.1186/s12885-015-1979-1

**Published:** 2015-12-16

**Authors:** Sangmi Kim, David L. Shore, Lauren E. Wilson, Ethel I. Sanniez, Jae H. Kim, Jack A. Taylor, Dale P. Sandler

**Affiliations:** 1 Medical College of Georgia, Department of Medicine—Section of Hematology/Oncology, Augusta University GRU Cancer Center, 1410 Laney Walker Blvd., Augusta, GA 30912 USA; 2 Westat, Durham, NC 27703 USA; 3 Epidemiology Branch, National Institute of Environmental Health Sciences, National Institutes of Health, Research Triangle Park, NC 27599 USA

**Keywords:** Breast cancer, Nonsteroidal anti-inflammatory drugs, Chemoprevention, Family history, Risk factor heterogeneity

## Abstract

**Background:**

Nonsteroidal anti-inflammatory drugs (NSAIDs) have been shown to inhibit several pathways in experimental models of breast carcinogenesis, but epidemiological evidence remains insufficient to support their use for breast cancer prevention. We examined the association between use of NSAIDs and breast cancer risk in a prospective cohort.

**Methods:**

The Sister Study is a prospective cohort study of women who had a sister(s) with breast cancer. As of December 2013, 2118 incident breast cancers were ascertained from 50,884 women enrolled between 2003 and 2009. Lifetime history of NSAID use was estimated from self-reported data in pill-years, with 1 pill per week for a year equivalent to 1 pill-year. Cox regression models were used to estimate hazard ratios (HRs) of breast cancer in relation to pill-years of use for different NSAIDs, with adjustment for potential confounders.

**Results:**

In the full cohort, although there was some evidence that use of non-aspirin, non-COXib NSAIDs was associated with lower breast cancer risk, there was little evidence of overall association for most categories of NSAID use. Among postmenopausal women NSAID use was not associated with reduced risk of breast cancer. However, among premenopausal women there was significantly reduced risk for any NSAID (HR_4vs1_ = 0.66, 95 % CI: 0.50–0.87) and specifically for aspirin (HR_4vs1_ = 0.57, 95 % CI: 0.33–0.98), with small, but non-significant reductions in risk for other drug classes.

**Conclusion:**

Women with a sister with breast cancer are themselves at increased risk and might benefit the most from chemoprevention. Although there was little evidence of protective effect from NSAIDs in the overall cohort of women or among the subset who are postmenopausal, there is intriguing evidence that NSAID use, particularly aspirin, may reduce risk among premenopausal women.

**Electronic supplementary material:**

The online version of this article (doi:10.1186/s12885-015-1979-1) contains supplementary material, which is available to authorized users.

## Background

Nonsteroidal anti-inflammatory drugs (NSAIDs) are a class of drugs commonly used to treat pain, fever and inflammation [1]. Based on their long-term safety and preliminary efficacy data, there has been interest in aspirin and other NSAIDs as possible candidates for breast cancer prevention [2]. NSAIDs exert pharmacological effects primarily by blocking cyclooxygenase (COX) enzyme activity to suppress the prostaglandin synthesis pathway [1, 3]. COX-independent effects of NSAIDs have been described, but the chemopreventive potential of NSAIDs is thought to be closely related to their inhibition of the COX-2 enzyme [4]. Overexpression of COX-2 has been implicated in various processes related to breast carcinogenesis such as inhibition of apoptosis, promotion of angiogenesis, and impairment of immune surveillance [3]. Notably, upregulation of PGE_2,_ the most abundant COX-2—derived prostaglandin [5], can also induce *CYP19* transcription and increase aromatase activity, leading to increased estrogen production in mammary adipose tissue [6].

Numerous epidemiologic studies have investigated the relation between use of NSAIDs and breast cancer. Although previous meta-analyses have suggested a modest reduction in breast cancer risk in relation to use of aspirin and other NSAIDs [7, 8], a randomized clinical trial of low dose aspirin [9] and recent analyses of large cohort studies [10, 11] do not support a protective association. Moreover, results concerning the influence of frequency, duration or types of NSAIDs are largely inconsistent even within studies that found an inverse association between use of NSAIDs and breast cancer [12–15]. Assessment and quantification of NSAID use are challenging in epidemiologic studies. Except for a few studies based on healthcare databases which have their own limitation in assessing over-the-counter NSAIDs [16, 17], information on use of NSAIDs is collected using questionnaires that vary across studies, possibly accounting for inconsistent results.

To further clarify the role of NSAID use in breast cancer prevention, we examined the association between lifetime use of NSAIDs and breast cancer risk in a large prospective cohort of women with a sister with breast cancer.

## Methods

### Sister study

The Sister Study (NCT00047970) is a prospective cohort of 50,884 women aged 35–74 years who had a sister with breast cancer but who did not have breast cancer themselves [18, 19]. They were recruited through various sources across the United States and Puerto Rico between 2003 and 2009. Eligible participants completed a telephone interview that sought information on reproductive and medical history, environmental and occupational exposures, and a number of lifestyle factors, and completed a home visit during which blood and other biological and environmental specimens were collected and height, weight, waist circumference and blood pressure were measured. Participants are followed annually to update contact information and report diagnosis of selected medical conditions including breast cancer. Participants complete a more comprehensive questionnaire about changes in exposures and health status every 2–3 years. Response rates over time have been consistently high; 92 % of participants completed their most recent scheduled follow-up activity (annual update or comprehensive questionnaire). The study was approved by the Institutional Review Board of the National Institute of Environmental Health Sciences, NIH and the Copernicus Group Institutional Review Board, and all participating women gave written informed consent.

### Assessment of NSAID use

Information on NSAID use was obtained during the baseline interview in multiple ways. Prior to telephone interview, study participants were given a medications booklet listing common medications and were told to use them as a reference to identify specific medications that they used. During the computer assisted telephone interview, participants were first asked about medications used for 25 major health conditions including some for which NSAIDs may be indicated (e.g. several cardiovascular diseases, rheumatoid arthritis and migraines). Participants were asked the total number of years they took any medication for a specific condition and the names of each medication currently taken at least once a week for that condition. For each medication reported they were asked to report the age at first use, the number of days per week, times per day on days they took it, and total years or months of use. If the medications reported did not span at least half of the interval between first use of medicine for a condition and the date of interview, participants were asked for similar information for the medication they took for that condition for the longest time. Participants were also asked about any medications they were currently taking at least once a week that were not covered in the medical history section of the questionnaire. Finally, participants were asked if they had ever taken any pain or inflammation medications at least three times a week for 3 months in a row or longer that might not have been reported elsewhere in the interview. Again, participants were asked to report age at first use, whether they had used it regularly in the past 12 months, how many days per week they usually took the medication, and how many times a day they took the medication on days that they used it.

Each reported medication was coded by product and class using the Slone Drug Dictionary [20]. Products that contained more than one active ingredient were assigned multiple class codes. We grouped aspirin, acetyl salicylate and products containing these drugs as “aspirin”, and grouped celecoxib, rofecoxib and valdecoxib as selective COX-2 inhibitors (COXibs). We used the term non-aspirin, non-COXib NSAIDs to denote NSAIDs that did not belong to either the aspirin or the COXibs group. These included agents such as proprionic acids (e.g., ibuprofen, naproxen), indole (e.g., sulindac) and enolic acids (e.g., piroxicam). Aspirin, COXibs and non-aspirin, non-COXib NSAIDs are collectively referred to as NSAIDs.

Using the detailed medication use histories provided, we constructed age-specific calendar grids for use of each specified NSAID. Total years of use for each subgroup of NSAIDs were computed by summing across the occupied ages in the calendar grid. To quantify lifetime use of NSAIDs, the frequency of use during the period was entered into the corresponding age grids and pill-years of use were computed as a product of frequency and years of use, where one pill-year represents use of one pill per week for a year.

### Documentation of breast cancer diagnosis

Participants have been followed through 2013 with an average of 5.3 years of follow-up. As of December 26, 2013, a breast cancer diagnosis was documented in 2118 women in the cohort, with an average time to diagnosis of 3.2 years. Women who report a new breast cancer diagnosis were contacted 6 months after diagnosis for additional information about their diagnosis and treatment and asked to authorize release of pertinent medical records. To date, pathology reports or complete medical records have been obtained for 77 % of self-reported breast cancers (*N* = 1638). Because of high agreement between self-reports and medical records (from 91.5 % for human epidermal growth factor receptor 2 [HER2] status to 99.5 % for any breast cancer diagnosis, invasive breast cancer, and estrogen receptor positive [ER+] invasive cancer), we retained self-reported breast cancers in this analysis.

### Data analysis

Cox proportional hazard models were used to estimate hazard ratios (HR) and 95 % confidence intervals (CI) for the associations between lifetime use of NSAIDs and breast cancer risk. Pill-years of NSAID use was categorized into 4 groups: <0.75 pill-years (equivalent to less than 3 pills/week for 3 months); 0.75- < 14 pill-years; 14- < 49 pill-years; and ≥ 49 pill-years to reflect similar levels of frequency and duration of use that were used to define use of NSAIDs in previous studies [21, 22]. The highest exposure category can be achieved in several ways, including by taking 1 NSAID pill/day for 7 years or by taking 1 pill/week for 49 years. The primary time scale was age, and women were delay-entered into risk sets (left-truncated) beginning at the age when they completed baseline activities. Person-time accrued until date of diagnosis for incident breast cancers, or the most current follow-up questionnaire completion date for the rest of the cohort who were up to date in their follow-up cycle. Women who did not respond to their most recent eligible health update were censored at the earliest date among final non-response, death, or the midpoint of the interval between the last completed health update and the end of the window of eligibility for responding to their first skipped health update. Visual inspection did not suggest that the proportionality assumption was violated. Evaluation of interaction terms with a logarithmic function of time added to the Cox model further supported the proportionality assumption.

Models were adjusted for variables selected using a directed acyclic graph (DAG) analysis. Briefly, we postulated that “health consciousness” and “pain” were two immediate, but unmeasured, factors leading to use of NSAIDs, and considered variables that were related with breast cancer risk and at least one of the unmeasured variables, as potential confounders. The final adjusted models included: race/ethnicity (non-Hispanic white; black; Hispanic; or others), level of education (high school graduate or less; some colleges or associate degree; or college degree or higher), history of benign proliferative breast disease (fibrocystic/benign changes; fibroadenoma; proliferative changes; or ductal/lobular hyperplasia), number of 1^st^ degree family members with breast cancer (1; 2; or ≥ 3), BMI (<18.5; 18.5–24.9; 25–29.9; 30–34.9; 35–39.9; or ≥ 40 kg/m^2^), time since the last mammogram (<1 year; 1–< 2 years; or ≥ 2 years) and age at 1^st^ term birth (<24y; 24–29y; ≥ 30y; or nulliparous). Additional adjustment for years of oral contraceptive use, alcohol intake, current physical activity and years of hormone replacement therapy (HRT) use did not change the results from the specified multivariable models (Additional file 1: Table S1).

In addition to an analysis including all incident breast cancers, we conducted *a priori* subgroup analyses by tumor characteristics by treating tumor occurrences that were assigned to types not under consideration as censoring events. Specifically, we evaluated whether an association with NSAID use might vary between invasive versus *in situ* tumors, or according to estrogen receptor (ER), progesterone receptor (PR) and HER2 status. Prespecified (menopausal status, BMI and number of 1^st^ degree family members with breast cancer) and data-driven *post hoc* (parity and age at first birth) subgroup analyses were also performed. Differences in the HRs between levels of these factors were evaluated by testing for multiplicative interactions or for homogeneity of trends on exposure categories as an ordinal variable. Significance tests were two-sided with the level of significance at 0.05. Stata 12.1 (College Station, TX) was used for all analyses.

## Results

Approximately 11 % of premenopausal women and 21 % of postmenopausal women had at least 49 pill-years of NSAID use in their lifetime (Table 1). High NSAID use was associated with older age, non-Hispanic white race and higher BMI. History of benign proliferative breast disease and other medical conditions managed with NSAIDs was more common among the high NSAID user group. Among premenopausal women, the high NSAID users were also more likely to be current smokers.

**Table 1:** Characteristics of study participants according to NSAID use and menopause status, the Sister Study (2003-2013)

	All	Premenopausal women		Postmenopausal women	
Lifetime use of NSAIDs		< 49 pill-years^a^	≥ 49 pill-years^a^	< 49 pill-years^a^	≥ 49 pill-years^a^
No.	50,883	16,311	1960	25,590	6860
*Mean (SD)*					
Age, y	55.2 (9.0)	46.4 (5.2)	48.3 (5.0)	59.6 (6.8)	61.5 (6.6)
BMI, kg/m^2^	27.8 (6.3)	27.3 (6.3)	29.2 (7.2)	27.7 (6.0)	29.1 (6.7)
Had alcoholic drinks, days/week	1.5 (2.1)	1.4 (1.9)	1.5 (2.0)	1.6 (2.2)	1.6 (2.3)
Age at menarche, y	12.6 (1.5)	12.8 (1.5)	12.6 (1.6)	12.6 (1.5)	12.5 (1.5)
Duration of HRT use, y	3.8 (6.6)	0.4 (1.7)	0.8 (2.8)	5.3 (7.3)	7.2 (8.1)
*No. (%)*					
Non-Hispanic white	42,558 (83.7)	12,931 (80.1)	16,480 (85.5)	21,490 (84.0)	6190 (90.3)
Having ≥ college degree	25,886 (50.9)	9090 (55.8)	956 (48.8)	12,505 (48.9)	3244 (47.8)
Current smokers	4175 (8.2)	1424 (8.7)	254 (13.0)	1974 (7.7)	510 (7.4)
Nulliparity	9207 (18.1)	3365 (20.6)	478 (21.9)	4240 (16.6)	1143 (16.7)
Current use of HRT	5276 (10.4)	536 (3.3)	136 (7.0)	3478 (13.7)	1111 (16.3)
Having ≥ 2 first degree family members with breast cancer	13,275 (27.0)	3544 (21.7)	406 (23.8)	6836 (26.7)	1893 (27.6)
History of benign proliferative breast disease^b^	1842 (3.6)	642 (3.9)	99 (5.1)	827 (3.2)	240 (3.5)
Prior diagnosis of major medical conditions^c^	16,408 (32.3)	4464 (27.4)	920 (46.9)	7837 (30.6)	3129 (45.6)
Having a mammogram within a year	40,928 (81.4)	12,695 (79.7)	1537 (79.4)	20,857 (82)	5712 (83.6)

^a^One pill-year is equivalent to taking 1 pill per week for 1 year

^b^Diagnosis of fibrocystic or benign changes, fibroadenoma, proliferative changes, ductal or lobular hyperplasia

^c^Medical conditions associated with use of NSAIDs including angina, heart attack, stroke, ischemic heart attack, rheumatoid arthritis, migraine and scoliosis

There were no significant associations between use of any NSAID and breast cancer risk, when analyzing duration and frequency separately (Table 2). However, for pill-years of NSAID use, which is a combined measure of duration and frequency, the highest category of non-aspirin, non-COXib NSAIDs was associated with reduced risk compared to the lowest category (multivariable-adjusted HR = 0.84, 95 % CI: 0.71–0.99). Neither pill-years of aspirin nor pill-years of COXibs were inversely associated with breast cancer risk. Results were similar for low- and regular-dose aspirin use and these were not evaluated separately in subsequent analyses.

**Table 2:** Lifetime use of different NSAIDs and breast cancer risk according to duration, average frequency and pill-years^a^ of use, the Sister Study (2003–2013)

	Cases	HR (95 % CI)^b^		Cases	HR (95 % CI)^b^		Cases	HR (95 % CI)^b^
*Aspirin*								
*Duration of use, y*			*Frequency of use, per week*			*Pill-years of use, py*		
<5	1830	1. (Ref.)	No use	1483	1. (Ref.)	<0.75	1489	1. (Ref.)
5- < 10	132	0.83 (0.69–1.02)	<4	71	0.87 (0.68–1.13)	0.75- < 14	191	1.09 (0.94–1.28)
10- < 20	97	0.95 (0.76–1.19)	4- < 7	82	1.14 (0.9–1.43)	14- < 49	266	0.97 (0.85–1.11)
≥ 20	59	0.93 (0.70–1.24)	≥ 7	481	1.07 (0.95–1.21)	≥ 49	172	0.95 (0.81–1.12)
*P* ^c^		0.386	*P* ^c^		0.202	*P* ^c^		0.593
*Low dose aspirin*								
*Duration of use, y*			*Frequency of use, per week*			*Pill-years of use, py*		
<1	1978	1. (Ref.)	No use	1708	1. (Ref.)	<0.75	1711	1. (Ref.)
1- < 5	90	0.86 (0.72–1.11)	<7	61	0.99 (0.76–1.29)	0.75- < 21	203	1.07 (0.92–1.24)
≥ 5	50	0.88 (0.65–1.2)	≥ 7	349	1.05 (0.91–1.2)	≥ 21	204	0.93 (0.8–1.08)
*P* ^c^		0.240	*P* ^c^		0.539	*P* ^c^		0.541
*Regular strength aspirin*								
*Duration of use, y*			*Frequency of use, per week*			*Pill-years of use, py*		
<1	1965	1. (Ref.)	No use	1866	1. (Ref.)	<0.75	1870	1. (Ref.)
1- < 5	45	0.76 (0.55–1.06)	<7	94	0.99 (0.77–1.26)	0.75- < 21	92	0.98 (0.79–1.21)
≥ 5	108	0.98 (0.77–1.25)	≥ 7	156	1.12 (0.92–1.36)	≥ 21	156	1.03 (0.87–1.22)
*P* ^c^		0.726	*P* ^c^		0.590	*P* ^c^		0.775
*COXibs* ^*d*^								
*Duration of use, y*			*Frequency of use, per week*			*Pill-years of use, py*		
<1	1954	1. (Ref.)	No use	1901	1. (Ref.)	<0.75	1904	1. (Ref.)
1- < 5	134	1.06 (0.76–1.43)	<7	42	1.19 (0.82–1.72)	0.75- < 21	139	1.05 (0.88–1.25)
≥ 5	30	0.71 (0.46–1.09)	≥ 7	175	0.95 (0.73–1.24)	≥ 21	75	0.86 (0.68–1.09)
*P* ^c^		0.205	*P* ^c^		0.590	*P* ^c^		0.431
*Non-aspirin, non-COXib NSAIDs*								
*Duration of use, y*			*Frequency of use, per week*			*Pill-years of use, py*		
<5	1845	1. (Ref.)	No use	1462	1. (Ref.)	No use	1470	1. (Ref.)
5- < 10	128	1.05 (0.86–1.28)	<4	156	1.04 (0.87–1.26)	<4	248	0.94 (0.85–1.11)
10- < 20	99	0.85 (0.7–1.06)	4- < 7	130	1.13 (0.93–1.37)	4- < 7	241	1.02 (0.89–1.17)
≥ 20	46	0.92 (0.68–1.25)	≥ 7	369	0.90 (0.79–1.02)	≥ 7	159	0.84 (0.71–0.99)
*P* ^c^		0.260	*P* ^c^		0.144	*P* ^c^		0.130

^a^One pill-year is equivalent to taking 1 pill per week for 1 year

^b^Adjusted for race/ethnicity (non-Hispanic white; black; Hispanic; or others), level of education (high school graduate or less; some colleges or associate degree; or college degree or higher), history of benign proliferative breast disease (fibrocystic/benign changes; fibroadenoma; proliferative changes; or ductal/lobular hyperplasia), number of 1^st^ degree family members with breast cancer (1; 2; or ≥ 3), BMI (<18.5; 18.5–24.9; 25–29.9; 30–34.9; 35–39.9; or ≥ 40 kg/m^2^), age at 1^st^ term birth (<24y; 24–29y; ≥ 30y; or nulliparous), time since the last mammogram (<1 year; 1- < 2 years; or ≥ 2 years) and menopause status at diagnosis; duration and frequency of use were mutually adjusted

^c^*P* for linear trend

^d^Selective COX-2 inhibitors such as celecoxib, rofecoxib or valdecoxib

Approximately 26 % of all incident breast cancers were *in situ* tumors, and of the remaining invasive cases, 1203 women were diagnosed at stages I or II, and 216 women were diagnosed at later stages (stage III or IV). Associations with NSAID use did not differ among *in situ* tumors, localized and advanced breast cancers (Additional file 1: Table S2). Invasive breast cancers were also categorized based on ER, PR and HER2 status (Table 3). *In situ* tumors were not included in this analysis because they are not routinely evaluated for these molecular markers. The majority (75 %) of invasive cancers were ER+/PR+/HER2-, followed by ER-/PR-/HER2- (11 %) and ER+/PR+/HER2+ (10 %). There were too few ER-/PR-/HER2+ tumors to evaluate (*N* = 53). There was an indication of inverse associations between long-term regular use of any NSAIDs and ER+/PR+ breast cancer regardless of HER2 status. While most associations were not statistically significant, there was a significant inverse trend for increasing pill years of non-aspirin, non-COXib NSAIDs and ER+/PR+/HER2- breast cancer (*p* = 0.039). The relationship between use of NSAIDs and breast cancer risk did not vary by degree of family history of breast cancer or BMI (data not shown).

**Table 3:** Associations between pill-years^a^ of NSAID use and invasive breast cancer by estrogen receptor (ER), progesterone receptor (PR) and HER2 status, the Sister Study (2003–2013)

	No. Cases	HR (95 % CI)^c^	No. Cases	HR (95 % CI)^c^	No. Cases	HR (95 % CI)^c^
	*ER+/PR+/HER2-*		*ER+/PR+/HER2+*		*ER-/PR-/HER2-*	
Use of any NSAIDs, py						
<0.75	467	1. (Ref.)	64	1. (Ref.)	56	1. (Ref.)
0.75- < 14	156	1.05 (0.88–1.27)	15	0.72 (0.41–1.26)	26	1.40 (0.87–2.24)
14- < 49	194	0.94 (0.79–1.11)	28	0.96 (0.61–1.51)	28	1.20 (0.76–1.90)
≥ 49	171	0.85 (0.7–1.01)	25	0.87 (0.54–1.4)	26	1.22 (0.75–1.97)
P for trend		0.073		0.624		0.374
Aspirin, py						
<0.75	682	1. (Ref.)	97	1. (Ref.)	95	1. (Ref.)
0.75- < 14	102	1.25 (1.01–1.54)	8	0.68 (0.33–1.41)	17	1.07 (0.57–2.01)
14- < 49	118	0.89 (0.73–1.09)	16	0.85 (0.5–1.47)	22	1.43 (0.88–2.3)
≥ 49	86	0.97 (0.77–1.22)	11	0.9 (0.47–1.7)	11	1.15 (0.61–2.19)
P for trend		0.569		0.501		0.261
COXibs^b^, py						
<0.75	890	1. (Ref.)	118	1. (Ref.)	122	1. (Ref.)
0.75- < 21	67	1.06 (0.83–1.36)	8	0.92 (0.45–1.89)	11	1.36 (0.73–2.53)
≥ 21	31	0.74 (0.51–1.06)	6	1.02 (0.44–2.33)	6	1.16 (0.51–2.66)
P for trend		0.238		0.942		0.44
Non-aspirin, non-COXib NSAIDs, py						
<0.75	703	1. (Ref.)	89	1. (Ref.)	93	1. (Ref.)
0.75- < 14	112	0.93 (0.76–1.13)	19	1.17 (0.71–1.93)	21	1.25 (0.77–2.03)
14- < 49	96	0.84 (0.68–1.05)	14	0.9 (0.51–1.59)	14	0.93 (0.53–1.63)
≥ 49	77	0.83 (0.65–1.05)	10	0.77 (0.4–1.5)	11	0.94 (0.44–1.77)
P for trend		0.039		0.503		0.892

^a^One pill-year is equivalent to taking 1 pill per week for 1 year

^b^Selective COX-2 inhibitors such as celecoxib, rofecoxib or valdecoxib

^c^Adjusted for race/ethnicity (non-Hispanic white; black; Hispanic; or others), level of education (high school graduate or less; some colleges or associate degree; or college degree or higher), history of benign proliferative breast disease (fibrocystic/benign changes; fibroadenoma; proliferative changes; or ductal/lobular hyperplasia), number of 1^st^ degree family members with breast cancer (1; 2; or ≥ 3), BMI (<18.5; 18.5–24.9; 25–29.9; 30–34.9; 35–39.9; or ≥ 40 kg/m^2^), age at 1^st^ term birth (<24y; 24–29y; ≥ 30y; or nulliparous), time since the last mammogram (<1 year; 1- < 2 years; or ≥ 2 years) and menopause status at diagnosis

In contrast to results for postmenopausal women, lifetime use of NSAIDs was associated with reduced risk of breast cancer among premenopausal women (Table 4). The multivariable-adjusted HRs for the highest versus the lowest category of pill-years of NSAID use were 0.66 (95 % CI: 0.50–0.87) among premenopausal women versus 0.95 (95 % CI: 0.82–1.09) among postmenopausal women. The heterogeneous associations between use of NSAIDs and breast cancer risk by menopause status were most apparent for aspirin (*p* for homogeneity of trends = 0.038): The HRs associated with the highest category of pill-years of aspirin use were 0.57 (95 % CI: 0.33–0.98) in premenopausal women and 1.03 (95 % CI: 0.87–1.23) among postmenopausal women. Although results were not statistically significant, the inverse associations with the highest category of pill-years of use of COXibs and other non-aspirin, non-COXib NSAIDs also tended to be more evident among premenopausal women.

**Table 4:** Associations between pill-years^a^ of NSAID use and breast cancer by menopause status, the Sister Study (2003-2013)

	No. Cases	HR (95 % CI)^c^	No. Cases	HR (95 % CI)^c^	P for test of homogeneity of trends
	*Premenopausal women*		*Postmenopausal women*		
Use of any NSAIDs, py					0.091
<0.75	431	1. (Ref.)	576	1. (Ref.)	
0.75- < 14	91	0.84 (0.67–1.05)	220	1.07 (0.92–1.25)	
14- < 49	113	1.03 (0.83–1.27)	329	1.05 (0.91–1.20	
≥ 49	60	0.66 (0.50–0.87)	298	0.95 (0.82–1.09)	
P for trend		0.025		0.641	
Aspirin, py					0.038
<0.75	606	1. (Ref.)	883	1. (Ref.)	
0.75- < 14	35	0.81 (0.58–1.14)	156	1.21 (1.02–1.44)	
14- < 49	40	0.91 (0.66–1.25)	226	1.00 (0.86–1.16)	
≥ 49	14	0.57 (0.33–0.98)	158	1.03 (0.87–1.23)	
P for trend		0.038		0.654	
COXibs^b^, py					0.200
<0.75	659	1. (Ref.)	1245	1. (Ref.)	
0.75- < 21	27	0.88 (0.60–1.30	112	1.09 (0.90–1.32)	
≥ 21	9	0.62 (0.32–1.22)	66	0.91 (0.71–1.17)	
P for trend		0.141		0.812	
Non-aspirin, non-COXib NSAIDs, py					0.508
<0.75	497	1. (Ref.)	973	1. (Ref.)	
0.75- < 14	78	0.93 (0.73–1.18)	170	1.00 (0.85–1.18)	
14- < 49	79	1.04 (0.82–1.33)	162	1.00 (0.85–1.19)	
≥ 49	41	0.73 (0.53–1.01)	118	0.87 (0.72–1.06)	
P for trend		0.182		0.314	

^a^One pill-year is equivalent to taking 1 pill per week for 1 year

^b^Selective COX-2 inhibitors such as celecoxib, rofecoxib or valdecoxib

^c^Adjusted for race/ethnicity (non-Hispanic white; black; Hispanic; or others), level of education (high school graduate or less; some colleges or associate degree; or college degree or higher), history of benign proliferative breast disease (fibrocystic/benign changes; fibroadenoma; proliferative changes; or ductal/lobular hyperplasia), number of 1^st^ degree family members with breast cancer (1; 2; or ≥ 3), BMI (<18.5; 18.5–24.9; 25–29.9; 30–34.9; 35–39.9; or ≥ 40 kg/m^2^), time since the last mammogram (<1 year; 1- < 2 years; or ≥ 2 years) and age at 1^st^ term birth (<24y; 24–29y; ≥ 30y; or nulliparous)

Several exploratory analyses were conducted to better understand the unexpected subgroup finding. First, our analysis used age as the time scale for calculating HRs rather than time since enrollment, based on the results from a simulation study of different time scales in Cox regression analysis [23]. Since women were required to be breast cancer free at enrollment, those who enrolled at later ages could have a lower hazard than those of the same age who were enrolled at younger ages [24]. However, additional adjustment for age at study entry had negligible impact on the risk estimates for pre- and post-menopausal women.

Second, median pill-years of NSAID use were lower for premenopausal than for postmenopausal women (18.7 vs 28 years). To make the analyses in the two groups more comparable, we further divided the highest category of NSAID use (≥21 pill-years for COXibs and ≥ 49 pill-years for other types of NSAIDs) into two groups using the median among postmenopausal women (not shown), but the association with breast cancer risk remained unchanged (HR for ≥ 91 versus <0.75 pill-years of NSAID use = 1.01, *p* = 0.89 in postmenopausal women). We also examined breast cancer risk in relation to pill-years of NSAID use within the past 5 years (Additional file 1: Table S3). Again, the results were similar, suggesting an inverse, albeit not significant, association between use of different NSAIDs and breast cancer risk only among premenopausal women (HR for ≥ 14 versus <0.75 pill-years of NSAID use = 0.84, *p* = 0.09 in premenopausal women vs. 1.02, *p* = 0.78 in postmenopausal women). Third, separate analysis, stratified by menopause status at study follow-up, was conducted because 46 % of premenopausal women at baseline (including 30 % of premenopausal women who developed breast cancer) became postmenopausal by the end of study follow-up. This analysis did not affect the lack of the association among postmenopausal women: the HRs for the highest pill-years of aspirin were 0.55 (95 % CI: 0.24–1.23) vs 0.99 (95 % CI:0.83–1.17) for those who were premenopausal versus postmenopausal at follow-up (p for homogeneity of trends =0.067).

Lastly, we performed *post-hoc* analysis to explore the influence of other reproductive factors. Results stratified by parity suggested stronger inverse associations between high pill-years of non-aspirin, non-COXib NSAID use and breast cancer risk among nulliparous premenopausal women compared with among parous premenopausal women (*p* for interaction = 0.081; Additional file 1: Table S4). The inverse association between high pill-years of NSAID use and breast cancer risk was also more evident among premenopausal women who had not given birth by the age of 30, except for use of COXibs, but there were only a few women with incident breast cancer in this exploratory subgroup analysis. In postmenopausal women, we did not find evidence that these reproductive events modified the relationship between use of NSAIDs and breast cancer risk.

## Discussion

In this analysis of a prospective study of women with a sister with breast cancer, lifetime use of NSAIDs generally did not reduce the risk of developing breast cancer. However, among premenopausal women, our results suggest that lifetime use of different NSAIDs, particularly aspirin, may be associated with reduced breast cancer risk.

A wealth of experimental data supports the chemopreventive potential of NSAIDs. Although the mechanistic pathways are not well established, the primary mechanism appears to involve suppression of COX-2-PGE_2_ signaling, which can modulate cellular proliferation, survival and migration, and impair tumor immunosurveillance through multiple signaling pathways [3]. COX-2-PGE_2_ signaling has been directly implicated in breast cancer. Targeted overexpression of the COX-2 gene sufficiently induced mammary carcinogenesis and COX-2 inhibition reduced the incidence and extent of breast tumors in breast cancer animal models. [25–27]. PGE_2_ can also upregulate aromatase expression in stromal fat cells in the breast and consequently increase local estrogen production, suggesting that NSAIDs may be especially protective for ER+ breast cancer [28, 29]. However, there is also evidence from animal studies for the role of COX-2 in the development of ER- and HER2+ breast cancer through activation of Akt pathway [30].

There is yet insufficient epidemiological evidence to draw conclusions about the role of NSAIDs in breast cancer risk [2]. Overall, results of recent meta-analyses have concluded that aspirin and other NSAIDs might provide a slight reduction in breast cancer risk [7, 8, 31], but the inverse association tends to be more evident in case–control studies than in cohort studies [10, 15, 17, 32–34] or in the single randomized controlled trial of low-dose aspirin [9]. Although our study found some evidence that breast cancer risk was reduced among women with the highest lifetime use of non-aspirin, non-COXib NSAIDs, this finding was only significant when we categorized by pill-years of use and appears in association with tumors that are ER+/PR+/HER2-. In general, our findings are consistent with the previous prospective studies that did not support overall benefits of NSAID use for breast cancer prevention.

Some studies have suggested subgroup effects according to tumor and population characteristics. A stronger inverse association with NSAID use has been found for ER+ breast cancer in some [14, 35] but not all studies [12, 22, 36–38]. The limited available data, including ours, do not support different associations by HER2 status [38, 39]. An extended follow-up analysis of randomized clinical trials has suggested that daily aspirin use may reduce cancer mortality by reducing the incidence of metastatic cancer [40]. We are not yet able to study breast cancer mortality in our cohort. However, associations with use of NSAIDs were similar for *in situ*, localized and advanced breast cancer in our study.

Menopause status is considered an important effect modifier of several known breast cancer risk factors [41, 42]. We therefore evaluated the association between lifetime use of NSAIDs and breast cancer risk by menopause status and observed a protective association between high lifetime use of NSAIDs and breast cancer risk only among premenopausal women. Consistent with our findings, Zhang et al. reported that the odds ratios for regular use of NSAIDs were 0.62 (95 % CI: 0.41–0.94) among premenopausal women, but 0.90 (95 % CI: 0.69–1.16) in postmenopausal women in a large case–control study [22]. However, regular use of aspirin was similarly inversely associated with breast cancer regardless of menopause status in the Black Women’s Health Study [43], and no relationship between NSAID use and breast cancer was found among premenopausal women in the Nurses’ Health Study II [11]. Reasons for the inconsistent findings are difficult to pinpoint, but it is notable that our prospective study shares marked similarities to Zhang’s study [22] in terms of the assessment of NSAID use, where histories of medications were obtained through questions about an extensive list of major indications, and then detailed information for each episode of use was recorded. On the other hand, regular use (≥2 or ≥3 times per week) of aspirin and other NSAIDs was ascertained based on the baseline questionnaires, and the duration of use was estimated based on the responses to the same questions in each follow-up questionnaire in the aforementioned prospective cohort studies [11, 43].

We found that the protective association with NSAID use among premenopausal women was primarily restricted to the highest exposure category of 49 pill-years of NSAID use. The pill-year measure combines frequency and duration so the same number of pill-years can be derived from different combinations of frequency and duration of use, analogous to pack-years of smoking, a commonly used exposure measure. To address the possibility that older women may achieve the highest category by having more years of use but lower frequency of use, we further investigated the association between pill-years of NSAID use and breast cancer risk after dividing the highest exposure category into two groups (< 91 or ≥91 pill years). However, the HRs did not vary in the two strata.

Another possible explanation for the observed difference may lie in the interaction between NSAIDs and timing of exposure. The development of human breast tissue progresses over the lifespan, and menopause represents one lifecycle event associated with lobular involution and decrease in glandular or dense parenchymal features. Because hormone responsiveness and cell proliferation are the highest in the undifferentiated lobules [44–46], chemopreventive agents like NSAIDs may be more effective during the period susceptible to tumorigenesis. In the same context, we also evaluated other reproductive events affecting breast development such as parity and age at first term pregnancy. Our exploratory analysis suggested that premenopausal women who were nulliparous or did not give birth before age 30 years old might have greater benefit from lifetime use of NSAIDs. Further studies are necessary to determine whether women’s reproductive history can be used to inform clinical effectiveness of aspirin or other types of NSAIDs on breast cancer prevention.

Strengths of the present study include its prospective design, the large sample size and detailed information on potential risk factors for breast cancer. We also were able to characterize lifetime use of NSAIDs based on comprehensive questionnaire data about both medical history and medication use. In addition to questions about the general use of pain and inflammation medications, participants could report NSAID use in connection with medical histories, which included questions about all medications used in relation to 25 specific health conditions, and as part of a listing of any other medications that were currently being taken or were used within 1 year of enrollment. Despite various approaches (e.g., query of NSAID use prompted by specific medical conditions and visual cue cards for medications) used to improve the accuracy of recall, however, some nondifferential measurement error is unavoidable and is hard to quantify.

A few other issues concerning the interpretation of our study need to be discussed. First, we defined lifetime NSAID use based on the information collected at baseline and did not include information on changes in NSAID use during the study follow-up. However, given the relatively short time to breast cancer diagnosis in our study, it seems unlikely that change in NSAID use pattern is sufficiently large to influence the association between use of NSAIDs and breast cancer risk. Second, the present study involves multiple exposure and outcome measures, and the resulting larger number of statistical tests may have yielded spurious associations. Although the subgroup analyses were guided by biological plausibility and conducted in order to obtain a more comprehensive picture of the relationship between use of NSAIDs and breast cancer risk, findings from the subgroup analyses should be interpreted with caution. Lastly, our study population is unique in that all women had a sister with breast cancer at the time of enrollment. Because women with a family history of breast cancer on average have a 2–4 fold increased risk of developing breast cancer [47], women with an affected sister are a priority population for chemoprevention strategies. At the same time, the increased breast cancer risk in our cohort compared to the general population may also raise concern about potential lack of generalizability of findings. However, this increased risk is not due solely to rare high penetrance genes, but rather to possibly increased frequency of the same polymorphisms and environmental risk factors that affect most women. In fact, the prevalence of high penetrance breast cancer genes in our cohort was <1 % among those tested for BRCA1/BRCA2 mutations. Women in our study were similar to the general population in terms of prevalence of obesity, physical activity and other healthy lifestyle behaviors [48].

## Conclusion

In this analysis of more than 50,000 women at higher risk of breast cancer on the basis of family history, there was little evidence that use of NSAIDs can protect against breast cancer. However, among premenopausal women, use of NSAIDs, particularly aspirin, was associated with reduced risk of breast cancer, raising the intriguing hypothesis of chemopreventive benefit for this subgroup of at-risk women.

## Additional file

Additional file 1: Table S1. Comparison of HRs (95 % CIs) for breast cancer in relation to pill-years of use of different types of NSAIDs with additional adjustment for use of exogenous hormones, alcohol consumption and physical activity, the Sister Study (2003–2013). **Table S2.** Associations between pill-years of NSAID use and ductal carcinoma in situ versus invasive breast cancer, the Sister Study (2003–2013). **Table S3.** Associations between pill-years of NSAID use in the past 5 years and breast cancer risk by menopause status, the Sister Study (2003–2013). **Table S4.** High pill-years of NSAID use and breast cancer risk among premenopausal women by timing of reproductive events, the Sister Study (2003–2013). (DOCX 141 kb)

## Abbreviations

CIs: Confidence intervals
COX: Cyclooxygenase
DAG: Directed acyclic graph
ER: Estrogen receptor
HR: Hazard ratio
HER2: Human epidermal growth factor receptor 2
NSAIDs: Nonsteroidal anti-inflammatory drugs
PR: Progesterone Receptor
PGE_2_: Prostaglandin E_2_
COXibs: Selective COX-2 inhibitors
