# Supplementary material for: Lifetime use of nonsteroidal anti-inflammatory drugs and breast cancer risk: results from a prospective study of women with a sister with breast cancer
Source: BMC Cancer. 2015 Dec 16;15:960. doi: 10.1186/s12885-015-1979-1 (PMC4682256; doi:10.1186/s12885-015-1979-1)
Supplement: Additional file 1: Table S1. — Comparison of HRs (95 % CIs) for breast cancer in relation to pill-years of use of different types of NSAIDs with additional adjustment for use of exogenous hormones, alcohol consumption and physical activity, the Sister Study (2003–2013). Table S2. Associations between pill-years of NSAID use and ductal carcinoma in situ versus invasive breast cancer, the Sister Study (2003–2013). Table S3. Associations between pill-years of NSAID use in the past 5 years and breast cancer risk by menopause status, the Sister Study (2003–2013). Table S4. High pill-years of NSAID use and breast cancer risk among premenopausal women by timing of reproductive events, the Sister Study (2003–2013). (DOCX 141 kb) [file 12885_2015_1979_MOESM1_ESM.docx]

**Additional files 1: Table 1. Comparison of HRs (95% CIs) for breast cancer in relation to pill-years**^1^ **of use of different types of NSAIDs with additional adjustment**^2^ **for use of exogenous hormones, alcohol consumption and physical activity, the Sister Study (2003-2013)**

|  | All women | Premenopausal women | Postmenopausal women |
| --- | --- | --- | --- |
| Aspirin, py |  |  |  |
| <0.75 | 1. (Ref.) | 1. (Ref.) | 1. (Ref.) |
| 0.75-<14 | 1.11 (0.94-1.30) | 0.81 (0.57-1.15) | 1.24 (1.04-1.47) |
| 14-<49 | 0.96 (0.84-1.10) | 0.89 (0.64-1.24) | 0.99 (0.85-1.16) |
| ≥ 49 | 0.92 (0.78-1.09) | 0.45 (0.24-0.85) | 1.02 (0.85-1.22) |
| P for trend | 0.386 | 0.016 | 0.769 |
| COXibs ^3^, py |  |  |  |
| <0.75 | 1. (Ref.) | 1. (Ref.) | 1. (Ref.) |
| 0.75-<21 | 1. 06 (0.89-1.27) | 0.91 (0.62-1.35) | 1.11 (0.91-1.36) |
| ≥ 21 | 0.89 (0.7-1.13) | 0.59 (0.29-1.19) | 0.95 (0.74-1.23) |
| P for trend | 0.642 | 0.144 | 0.851 |
| Non-aspirin, non-COXib NSAIDs, py |  |  |  |
| <0.75 | 1. (Ref.) | 1. (Ref.) | 1. (Ref.) |
| 0.75-<14 | 1. 00 (0.94-1.30) | 0.93 (0.73-1.9) | 1.04 (0.88-1.22) |
| 14-<49 | 1.04 (0.84-1.10) | 1.01 (0.78-1.3) | 1.06 (0.9-1.26) |
| ≥ 49 | 0.82 (0.78-1.09) | 0.72 (0.52-1.01) | 0.86 (0.70-1.05) |
| P for trend | 0.164 | 0.144 | 0.467 |

^1^ One pill-year is equivalent to taking 1 pill per week for 1 year.

^2^ In addition to race/ethnicity (non-Hispanic white; black; Hispanic; or others), level of education (high school graduate or less; some colleges or associate degree; or college degree or higher), history of benign proliferative breast disease (fibrocystic/benign changes; fibroadenoma; proliferative changes; or ductal/lobular hyperplasia), number of 1^st^ degree family members with breast cancer (1; 2; or ≥ 3), BMI (<18.5; 18.5-24.9; 25-29.9; 30-34.9; 35-39.9; or ≥ 40 kg/m^2^), age at 1^st^ term birth (<24y; 24-29y; ≥ 30y; or nulliparous), time since the last mammogram (<1 year; 1-<2 years; or ≥ 2 years) and menopause status at diagnosis, the models were additionally adjusted for years of hormonal birth control use; total number of alcoholic drinks in the past year; total weekly metabolic hours across current physical activities; and years of estrogen/progestin use

^3^ Selective COX-2 inhibitors such as celecoxib, rofecoxib or valdecoxib

**Supplementary Table 2. Associations between pill-years^1^ of NSAID use and ductal carcinoma in situ versus invasive breast cancer, the Sister Study (2003-2013)**

|  | No. Cases | HR (95% CI)^3^ | No. Cases | HR (95% CI)^3^ | No. Cases | HR (95% CI)^3^ |
| --- | --- | --- | --- | --- | --- | --- |
|  | ***In Situ Cancer*** | | ***Localized breast cancer*** | | ***Advanced breast cancer*** | |
| Use of any NSAIDs, py |  |  |  |  |  |  |
| <0.75 | 242 | 1. (Ref.) | 556 | 1. (Ref.) | 107 | 1. (Ref.) |
| 0.75-<14 | 66 | 0.91 (0.69-1.2) | 193 | 1.09 (0.92-1.29) | 22 | 0.67 (0.42-1.07) |
| 14-<49 | 107 | 1.15 (0.91-1.45) | 238 | 0.97 (0.83-1.14) | 33 | 1.20 (0.85-1.69) |
| ≥ 49 | 77 | 0.89 (0.69-1.16) | 216 | 0.90 (0.77-1.06) | 34 | 0.84 (0.56-1.25) |
| P for trend |  | 0.868 |  | 0.239 |  | 0.820 |
| Aspirin, py |  |  |  |  |  |  |
| <0.75 | 354 | 1. (Ref.) | 836 | 1. (Ref.) | 157 | 1. (Ref.) |
| 0.75-<14 | 45 | 1.17 (0.86-1.6) | 114 | 1.14 (0.93-1.39) | 15 | 0.87 (0.51-1.49) |
| 14-<49 | 58 | 1.02 (0.77-1.35) | 150 | 0.93 (0.78-1.11) | 28 | 1.06 (0.7-1.61) |
| ≥ 49 | 35 | 0.94 (0.66-1.35) | 103 | 0.95 (0.77-1.18) | 16 | 0.95 (0.56-1.61) |
| P for trend |  | 0.966 |  | 0.53 |  | 0.963 |
| COXibs^2^, py |  |  |  |  |  |  |
| <0.75 | 440 | 1. (Ref.) | 1,076 | 1. (Ref.) | 200 | 1. (Ref.) |
| 0.75-<21 | 33 | 1.15 (0.81-1.64) | 88 | 1.15 (0.92-1.43) | 7 | 0.51 (0.24-1.09) |
| ≥ 21 | 19 | 1.08 (0.68-1.72) | 39 | 0.77 (0.55-1.06) | 9 | 1.00 (0.51-1.97) |
| P for trend |  | 0.507 |  | 0.44 |  | 0.395 |
| Non-aspirin, non-COXib NSAIDs, py |  |  |  |  |  |  |
| <0.75 | 336 | 1. (Ref.) | 835 | 1. (Ref.) | 149 | 1. (Ref.) |
| 0.75-<14 | 51 | 0.90 (0.67-1.2) | 152 | 0.98 (0.93-1.04) | 21 | 0.79 (0.5-1.26) |
| 14-<49 | 70 | 1.33 (1.02-1.73) | 116 | 0.68 (0.52-0.89) | 32 | 1.27 (0.86-1.88) |
| ≥ 49 | 35 | 0.86 (0.61-1.22) | 100 | 0.86 (0.61-1.2) | 14 | 0.73 (0.42-1.26) |
| P for trend |  | 0.705 |  | 0.185 |  | 0.700 |

^1^ One pill-year is equivalent to taking 1 pill per week for 1 year.

^2^ Selective COX-2 inhibitors such as celecoxib, rofecoxib or valdecoxib

^3^ Adjusted for race/ethnicity (non-Hispanic white; black; Hispanic; or others), level of education (high school graduate or less; some colleges or associate degree; or college degree or higher), history of benign proliferative breast disease (fibrocystic/benign changes; fibroadenoma; proliferative changes; or ductal/lobular hyperplasia), number of 1^st^ degree family members with breast cancer (1; 2; or ≥ 3), BMI (<18.5; 18.5-24.9; 25-29.9; 30-34.9; 35-39.9; or ≥ 40 kg/m^2^), age at 1^st^ term birth (<24y; 24-29y; ≥ 30y; or nulliparous), time since the last mammogram (<1 year; 1-<2 years; or ≥ 2 years) and menopause status at diagnosis.

**Supplementary Table 3. Associations between pill-years^1^ of NSAID use in the past 5 years and breast cancer risk by menopause status, the Sister Study (2003-2013)**

|  | No. Cases | HR (95% CI) ^3^ | No. Cases | HR (95% CI) ^3^ | No. Cases | HR (95% CI) ^3^ |
| --- | --- | --- | --- | --- | --- | --- |
|  | ***All*** | | ***Premenopausal women*** | | ***Postmenopausal women*** | |
| Use of any NSAIDs, py |  |  |  |  |  |  |
| <0.75 | 1,075 | 1. (Ref.) | 452 | 1. (Ref.) | 623 | 1. (Ref.) |
| 0.75-<14 | 356 | 1.01 (0.89-1.14) | 115 | 0.94 (0.76-1.16) | 241 | 1.05 (0.91-1.22) |
| ≥ 14 | 687 | 0.97 (0.87-1.07) | 128 | 0.84 (0.68-1.03) | 559 | 1.02 (0.90-1.14) |
| P for trend |  | 0.532 |  | 0.094 |  | 0.769 |
| Aspirin, py |  |  |  |  |  |  |
| <0.75 | 1,524 | 1. (Ref.) | 612 | 1. (Ref.) | 912 | 1. (Ref.) |
| 0.75-<14 | 204 | 1.08 (0.94-1.20) | 37 | 0.82 (0.59-1.15) | 167 | 1.19 (1.00-1.4) |
| ≥ 14 | 390 | 0.97 (0.88-1.09) | 46 | 0.80 (0.59-1.09) | 344 | 1.01 (0.89-1.15) |
| P for trend |  | 0.781 |  | 0.094 |  | 0.589 |
| COXibs^2^, py |  |  |  |  |  |  |
| <0.75 | 1,928 | 1. (Ref.) | 666 | 1. (Ref.) | 1,262 | 1. (Ref.) |
| 0.75-<14 | 100 | 1.10 (0.90-1.35) | 21 | 0.94 (0.61-1.45) | 79 | 1.15 (0.92-1.45) |
| ≥ 14 | 90 | 0.94 (0.76-1.16) | 8 | 0.50 (0.25-1.01) | 82 | 1.02 (0.82-1.28) |
| P for trend |  | 0.882 |  | 0.068 |  | 0.524 |
| Non-aspirin, non-COXib NSAIDs, py |  |  |  |  |  |  |
| <0.75 | 1,567 | 1. (Ref.) | 523 | 1. (Ref.) | 1,044 | 1. (Ref.) |
| 0.75-<14 | 277 | 1.00 (0.87-1.13) | 94 | 0.97 (0.78-1.22) | 183 | 1.01 (0.86-1.18) |
| ≥ 14 | 274 | 0.90 (0.79-1.03) | 78 | 0.90 (0.70-1.14) | 196 | 0.90 (0.75-1.97) |
| P for trend |  | 0.168 |  | 0.390 |  | 0.248 |

^1^ One pill-year is equivalent to taking 1 pill per week for 1 year.

^2^ Selective COX-2 inhibitors such as celecoxib, rofecoxib or valdecoxib

^3^ Adjusted for race/ethnicity (non-Hispanic white; black; Hispanic; or others), level of education (high school graduate or less; some colleges or associate degree; or college degree or higher), history of benign proliferative breast disease (fibrocystic/benign changes; fibroadenoma; proliferative changes; or ductal/lobular hyperplasia), number of 1^st^ degree family members with breast cancer (1; 2; or ≥ 3), BMI (<18.5; 18.5-24.9; 25-29.9; 30-34.9; 35-39.9; or ≥ 40 kg/m^2^), age at 1^st^ term birth (<24y; 24-29y; ≥ 30y; or nulliparous), time since the last mammogram (<1 year; 1-<2 years; or ≥ 2 years) and menopause status at diagnosis.

**Supplementary Table 4. High pill-years^1^** **of NSAID use and breast cancer risk among premenopausal women by timing of reproductive events, the Sister Study (2003-2013)**

|  | No. Patients | | HR (95% CI) ^3^ | No. Patients | HR (95% CI) ^3^ | P for interaction |
| --- | --- | --- | --- | --- | --- | --- |
|  | ***Parous women (N=14,468)*** | | | ***Nulliparous (N=3,793)*** | |  |
| Use of any NSAIDs, py |  | |  |  |  | 0.115 |
| < 49 | 481 | | 1. (Ref.) | 154 | 1. (Ref.) |  |
| ≥ 49 | 50 | | 0.77 (0.57-1.04) | 10 | 0.43 (0.22-0.85) |  |
| Aspirin, py |  | |  |  |  | 0.982 |
| < 49 | 520 | | 1. (Ref.) | 161 | 1. (Ref.) |  |
| ≥ 49 | 11 | | 0.58 (0.31-1.09) | 3 | 0.56 (0.18-1.77) |  |
| COXibs^2^, py |  | |  |  |  | 0.291 |
| < 21 | 523 | | 1. (Ref.) | 163 | 1. (Ref.) |  |
| ≥ 21 | 8 | | 0.77 (0.38-1.55) | 1 | 0.26 (0.04-1.86) |  |
| Non-aspirin, non-COXib NSAIDs, py |  | |  |  |  | 0.081 |
| < 49 | 496 | | 1. (Ref.) | 158 | 1. (Ref.) |  |
| ≥ 49 | 35 | | 0.87 (0.62-1.23) | 6 | 0.38 (0.16-0.93) |  |
|  | ***Gave birth before 30 years of age (N=10,460)*** | | | ***Had no birth by 30 years of age (N=7,785)*** | |  |
| Use of any NSAIDs, py |  |  | |  |  | 0.086 |
| < 49 | 325 | 1. (Ref.) | | 309 | 1. (Ref.) |  |
| ≥ 49 | 39 | 0.84 (0.80-1.17) | | 20 | 0.49 (0.31-0.78) |  |
| Aspirin, py |  |  | |  |  | 0.380 |
| < 49 | 355 | 1. (Ref.) | | 325 | 1. (Ref.) |  |
| ≥ 49 | 9 | 0.72 (0.37-1.4) | | 4 | 0.41 (0.15-1.09) |  |
| COXibs^2^, py |  |  | |  |  | 0.900 |
| < 21 | 359 | 1. (Ref.) | | 325 | 1. (Ref.) |  |
| ≥ 21 | 5 | 0.59 (0.29-1.44) | | 4 | 0.65 (0.24-1.76) |  |
| Non-aspirin, non-COXib NSAIDs, py |  |  | |  |  | 0.200 |
| < 49 | 337 | 1. (Ref.) | | 315 | 1. (Ref.) |  |
| ≥ 49 | 27 | 0.90 (0.61-1.34) | | 14 | 0.63 (0.31-0.93) |  |

^1^ One pill-year is equivalent to taking 1 pill per week for 1 year.

^2^ Selective COX-2 inhibitors such as celecoxib, rofecoxib or valdecoxib

^3^ Adjusted for race/ethnicity (non-Hispanic white; black; Hispanic; or others), level of education (high school graduate or less; some colleges or associate degree; or college degree or higher), history of benign proliferative breast disease (fibrocystic/benign changes; fibroadenoma; proliferative changes; or ductal/lobular hyperplasia), number of 1^st^ degree family members with breast cancer (1; 2; or ≥ 3), BMI (<18.5; 18.5-24.9; 25-29.9; 30-34.9; 35-39.9; or ≥ 40 kg/m^2^) and time since the last mammogram (<1 year; 1-<2 years; or ≥ 2 years).
